# Quinazoline Derivatives Designed as Efflux Pump Inhibitors: Molecular Modeling and Spectroscopic Studies

**DOI:** 10.3390/molecules26082374

**Published:** 2021-04-19

**Authors:** Ana-Maria Udrea, Andra Dinache, Jean-Marie Pagès, Ruxandra Angela Pirvulescu

**Affiliations:** 1 Laser Department, National Institute for Laser, Plasma and Radiation Physics, Magurele, 077125 Ilfov, Romania; ana.udrea@inflpr.ro; 2 Department of Anatomy, Animal Physiology and Biophysics, Faculty of Biology, University of Bucharest, 50095 Bucharest, Romania; 3 UMR-MD1, U1261, Aix-Marseille Univ, INSERM, SSA, MCT, 13385 Marseille, France; jean-marie.pages@univ-amu.fr; 4 Department of Ophthalmology, University of Medicine and Pharmacy Carol Davila, 020022 Bucharest, Romania; ruxandrapascu78@gmail.com; 5 Ophthalmology Clinic, Emergency University Hospital, 050098 Bucharest, Romania

**Keywords:** multidrug resistance (MDR), efflux pump inhibitor, AcrAB-TolC pump, quinazoline, molecular docking, UV-Vis spectroscopy, FTIR spectroscopy

## Abstract

Multidrug resistance of bacteria is a worrying concern in the therapeutic field and an alternative method to combat it is designing new efflux pump inhibitors (EPIs). This article presents a molecular study of two quinazoline derivatives, labelled BG1189 and BG1190, proposed as EPIs. *In silico* approach investigates the pharmacodynamic and pharmacokinetic profile of BG1189 and BG1190 quinazolines. Molecular docking and predicted ADMET features suggest that BG1189 and BG1190 may represent attractive candidates as antimicrobial drugs. UV-Vis absorption spectroscopy was employed to study the time stability of quinazoline solutions in water or in dimethyl sulfoxide (DMSO), in constant environmental conditions, and to determine the influence of usual storage temperature, normal room lighting and laser radiation (photostability) on samples stability. The effects of irradiation on BG1189 and BG1190 molecules were also assessed through Fourier-transform infrared (FTIR) spectroscopy. FTIR spectra showed that laser radiation breaks some chemical bonds affecting the substituents and the quinazoline radical of the compounds.

## 1. Introduction

The rise of multidrug resistance (MDR) of bacteria is a worldwide health problem, as hundreds of thousands of people die every year from bacterial infections. If actions are not taken, it is estimated that in less than 30 years, MDR bacteria will cause more deaths than cancer [1,2].

Different types of mechanisms are involved in the MDR acquired by bacteria: an accumulation of genes coding for single drug resistance or expelling medicines out of the bacteria through multidrug efflux pumps [3,4]. Efflux pumps are proteins located in bacterial membranes and their role is to extrude xenobiotics, preventing their accumulation inside the cells [5,6]. These transporters can mediate the bacterial resistance to a wide range of medicines. Efflux-pump mediated resistance is a major problem not only in fighting bacterial infections but also in the chemotherapy of cancer [7,8,9].

Considering the weak development of new antibiotics active in Gram-negative bacteria, alternative approaches in fighting resistance mechanisms must be investigated. One alternative is designing new chemosensitizers for combinational therapy with antibiotics which are sensitive to bacterial resistance process that can bypass the resistance mechanism and restore the bacterial susceptibility [10,11,12,13]. Over the years, several molecules were investigated with the purpose of inhibiting the efflux pumps of bacteria. First efflux pump inhibitor (EPI) described to inhibit multiple resistance-nodulation-division (RND) transporters from *Pseudomonas aeruginosa* was labelled MC-207,100 and now is known as PAβN (Phe-Arg-β-naphthylamide) [14,15]. Furthermore, a wide range of compounds was attested as EPIs. They can be natural, chemically synthesized, or semi-synthetic compounds, derived from existing EPIs [13]. The compounds studied in this article are part of the fully synthetic class.

Several series of quinazoline derivatives were tested for their ability to inhibit the activity of the efflux pumps in a panel of Gram-negative bacteria. Among them, 3-[3-(dimethylamino)propyl]-6-nitroquinazolin-4(3*H*)-one and 6-nitro-3-[2-(pyrrolidin-1-yl)ethyl]quinazolin-4(3*H*)-one, labelled BG1189 and BG1190, respectively, enhanced the activity of chloramphenicol against several Gram-negative isolates [16].

In this study, we evaluated the drug-likeness of quinazoline derivatives BG1189 and BG1190 by applying drug-like rules: Lipinski, Ghose and Veber [17,18,19]. Solubility, molecular weight or molar refractivity are properties that can indicate if a chemical compound has “drug-like” proprieties or not. Besides importance in “drug-like”, lipophilicity can be an indicator of antimicrobial activity. A coefficient of partition between octanol and water (log P*_o_*_/*w*_) with values between 1 and 2 is associated with high antimicrobial activity [20]. Pharmacokinetics of the two quinazoline derivatives were also predicted using *in silico* approaches.

Since these compounds are active on bacteria overexpressing the AcrAB–TolC pump, it is concluded that they inhibit the pump activity [16,21]. AcrB subunit of AcrAB–TolC drug export complex, is the active subunit of the complex and is well-characterized in Gram-negative bacteria [6,22].

To evaluate the mechanism of action of BG1189 and BG1190 we have used the crystal structure of the AcrB subunit from multidrug efflux pump (PDB code: 5NC5) from *Escherichia coli K-12* [23].

It was important to define the stability of the compounds before considering them for biomedical applications. The characteristics can vary under the influence of natural or artificial environmental factors and we should know for how long is it safe to keep them for a possible future utilization [24].

Time stability studies of quinazoline derivatives solutions were performed in constant environmental conditions (constant temperature and illumination), but also in variable environmental factors, analyzing the influence of usual storage temperature, normal room lighting and laser radiation (photostability). Photostability analyses first give information about the dose of radiation (normal room lighting or laser radiation) a medicine may be exposed to, without changing its properties and secondly may establish the illumination conditions needed to modify the molecules in order to create photoproducts with enhanced antimicrobial activity. Studies showed that exposing a medicine to UV-Vis radiation may alter its structure; the laser radiation, due to specific properties (wavelength and high energy), modifies medicines’ molecules in solutions, faster than incoherent radiation [25]. Laser modification of molecules and formation of derivatives was studied on different classes of compounds: phenothiazines [26,27,28,29,30], antibiotics [31], hydantoin derivatives [32,33], and ecdysteroids [34].

This study presents an overview of the spectral characteristics of two quinazoline derivatives previously designed as potential EPIs. It also intends to provide an insight into their stability and photostability in different environmental conditions. Moreover, using computational approaches, we evaluated the pharmacokinetic and pharmacodynamic profiles of quinazoline derivatives.

## 2. Results and Discussion

### 2.1. Pharmacokinetic Profile

BG1189 and BG1190 molecules fitted in with drug-like rules Lipinski, Ghose and Veber showed that both compounds respect all the applied rules. Based on these results, we can say that both BG1189 and BG1190 could be administrated as drugs. Drug-like rules validation for both derivatives and molecular descriptor predicted values are presented in Table 1.

We have analyzed ADMET features and compared them with norfloxacin. *In silico* ADMET results showed that BG1189 and BG1190 present high water solubility and high intestinal absorption, similar to norfloxacin.

Caco2 permeability reflected the ability of the compound to be absorbed by the intestinal cells. BG1189 and BG1190 permeability is lower than the antibiotic, but both derivatives have a higher predicted VDs’ value which indicates that they are expected to be distributed in tissue. Both molecules have a higher unbound fraction than norfloxacin, suggesting that these quinazoline derivatives diffuse or traverse easier in the cell membrane than norfloxacin. Total clearance of the new compounds is higher than norfloxacin total clearance. Norfloxacin and BG1189 are not Renal OCT2 substrate, so those drugs are not transported via OCT2. Regarding toxicity, BG1189, BG1190 and norfloxacin present similar hepatoxicity and rat LD50. BG1189 and BG1190 are both hERG II inhibitors (Table 2).

### 2.2. Pharmacodynamic Profile

#### 2.2.1. Lipophilicity Evaluation

BG1189 and BG1190 lipophilicity or hydrophobicity evaluation indicate a LogP(*_o/w_*) predicted value close to 1. This suggests a high antimicrobial activity of these compounds [20]. Values are presented in Table 1 as MlogP*_o/w_* and WlogP*_o/w_*.

#### 2.2.2. Molecular Docking

Low free energy of binding is correlated with high biological activity; a molecule is considered to have no biological activity if its binding energy is higher than −6 kcal/mol [35].

Molecular docking results indicate that the two quinazoline derivatives present a low estimated free energy of binding (EFEB) on the AcrB subunit of the multidrug efflux pump (Table 3).

**Figure 1:**
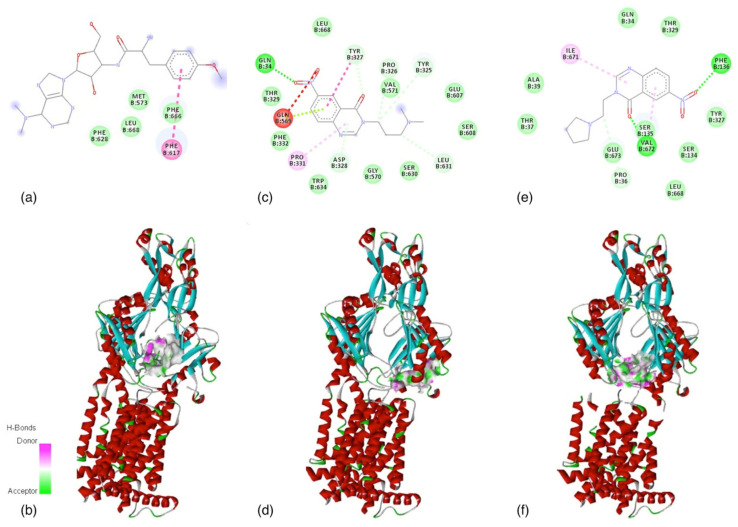
AcrB subunit of the multidrug efflux pump chain B (PDB code: 5NC5). (i) Experimental data—(**a**) AA in close contact with puromycin (antibiotic crystallized with the protein); (**b**) puromycin allosteric binding situs. (ii) Predicted data—(**c**,**e**) AA in close contact with BG1189 respectively BG1190; (**d**,**f**)—BG1189 respectively BG1190 interacting with pore domains of AcrB subunit. (**a**,**c**,**e**) Light green: van der Waals interactions; green: conventional hydrogen bond, grey: carbon hydrogen bond; red: unfavorable acceptor–acceptor interaction; dark pink: Pi–Pi T-shaped interaction; light pink: Pi–Alkyl interactions; (**b**,**d**,**f**) alpha helices of the protein are represented in red color, beta sheets in blue, turns in green, and random coil in grey. Images were obtained using Discovery Studio Visualizer [36].

The lowest EFEB is between BG1190 and the AcrB subunit, namely −8.21 kcal/mol (Figure 1e and Table 3). In both predicted cases, the compounds have a different binding situs from the allosteric one (Figure 1). The quinazoline derivatives are binding in the pocket domain [22] of AcrB, as well as puromycin antibiotic from the crystalized structure (Figure 1). This region actively presents the substrate to TolC tunnel for final expel [22].

### 2.3. Spectral Characterization

#### 2.3.1. Stability Study Using UV-Vis Absorption Spectroscopy 

**Figure 2:**
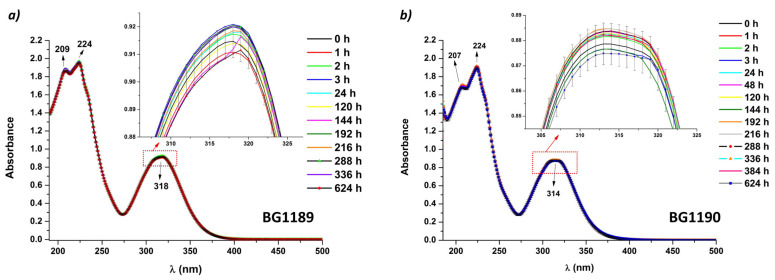
Time evolution of absorption spectra of quinazoline derivatives: (**a**) BG1189 and (**b**) BG1190. Solutions prepared in ultrapure water at 10^−3^ M; kept in dark, at 22 °C.

The stability of quinazoline derivatives was monitored under constant environmental conditions by recording the UV-Vis absorption spectra. The absorption spectra of the BG1189 and BG1190 solutions in ultrapure water at 10^−3^ M are presented in Figure 2.

Quinazoline derivatives’ solutions are highly stable in selected conditions for the incubation period (624 h), the difference in intensity between the absorption spectra remaining within the limits of error intervals. An extensive time stability study of a similar quinazoline derivative was performed in [37], indicating a high stability of the compound for 44 days, if kept in dark, at 4 °C.

In Figure 2a BG1189 exhibits three absorption peaks at 209, 224 and 318 nm. The absorption spectra of BG1190 (Figure 2b) are similar to the ones of BG1189, having the peaks at 207, 224 and 314 nm.

The absorption bands with maxima at 224 and 318/314 nm are representative for quinazolines and the absorption peak at 209/207 nm—a shoulder of 224 nm band—may be due to the influence of atmospheric oxygen.

The absorption band with the peak at 224 nm has a high absorbance and may originate from π–π* (1B) transitions. The absorption bands having the maximum at 318 nm for BG1189 and 314 nm for BG1190 may be assigned to π–π* (*^1^La* and *^1^Lb*) transitions. Additionally, the wide aspect of these absorption bands suggests a superposition with a band attributed to a *n*–π* transition [38].

Theoretical UV spectra and computations of the absorption spectrum of quinazolinone derivatives, having similar radicals to ours, assign the bands in 210–285 nm range to π–π* transitions, and the ones appearing in 285–320 nm to *n*–π* transitions. More so, the bands appearing at longer wavelengths were correlated to *n*–π* transition of the >C=O bonds coupled with the transition due to intramolecular charge transfer from phenyl ring and N=C–N bonds to >C=O group [39].

**Figure 3:**
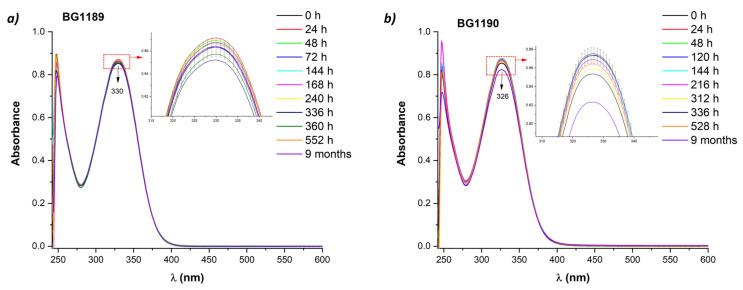
Time evolution of absorption spectra of quinazoline derivatives: (**a**) BG1189 and (**b**) BG1190. Solutions prepared in dimethyl sulfoxide (DMSO) at 10^−3^ M; kept in dark, at 22 °C.

Figure 3 displays the UV-Vis absorption spectra of the quinazoline derivatives in the range 250–600 nm (DMSO absorption is very strong below 250 nm saturating the detector).

BG1189 presents a maximal absorption band at 330 nm and BG1190 has a similar band with the peak at 326 nm. These bands, assigned to π–π* coupled with *n*–π* transitions, undergo a bathochromic shift compared to the absorption maxima of the quinazoline derivatives in water (318 nm for BG1189 and 314 nm for BG1190). Due to the solvent polarity, a blue shift appears with more polar solvent (from DMSO to water), which happens in the case of *n*–π* transitions [40]. This shift of the absorption maximum allows to identify that the dominant transitions responsible for the appearance of the longer wavelength peaks are *n*–π* transitions.

The long-term stability of the solutions in DMSO was assessed by comparing UV-Vis absorption spectra recorded periodically, from immediately after preparation up to 9 months.

For BG1189 solutions, the spectra remain stable up to 552 h (23 days) after preparation and this period may be considered as shelf life. On the other hand, BG1190 solutions in DMSO remain stable only for 216 h (9 days) after preparation. 

For both compounds, the stability is better in water solutions (624 h) than in DMSO.

Three temperatures were selected: 4 °C (refrigerator temperature), 22 °C (room temperature) and 37 °C (body temperature).

**Figure 4:**
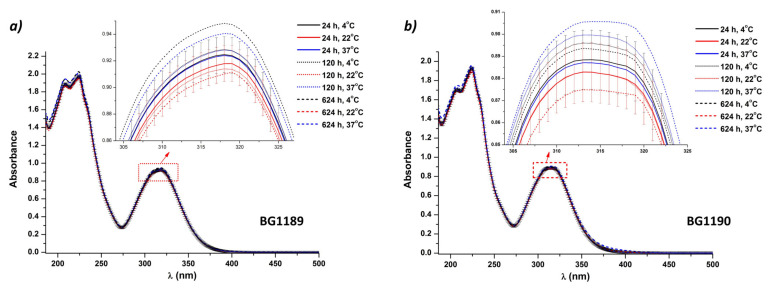
Influence of the storage temperature (4, 22 and 37 °C) on the absorption spectra of: (**a**) BG1189 and (**b**) BG1190. Solutions prepared in ultrapure water at 10^−3^ M, kept in dark.

From the grouping of the BG1189 absorption spectra, Figure 4a indicates that the most stable samples are observed at 22 °C. Even if before 120 h after preparation, all solutions are stable regarding the storage temperature, at the end of the experiments (624 h) the absorption spectra are outside the limits of the error bars. For potential biomedical applications, BG1189 in ultrapure water at 10^−3^ M should be preserved at 22 °C, if kept in dark, but no more the 624 h.

The absorption evolution of BG1190 solutions is similar to the one of BG1189 samples. The only difference observed in Figure 4b, is that the only spectrum that exceeds the error bars is the one recorded at 624 h for the BG1190 solution kept at 37 °C. The grouping of the absorption spectra for the entire time domain allows to conclude that BG1190 solutions have high stability, up to 624 h, if kept in dark at 4 °C or at 22 °C.

An important part of stability behavior for a new medicine is the evaluation of photostability.

**Figure 5:**
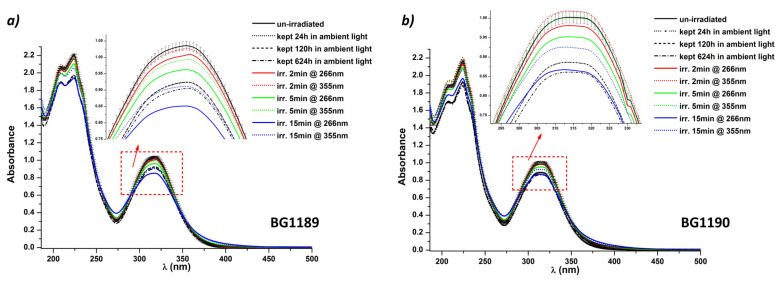
Influence of the illumination conditions on the absorption spectra of: (**a**) BG1189 and (**b**) BG1190. Solutions prepared in ultrapure water at 10^−3^ M, kept at 22 °C.

Figure 5 presents the influence of different illumination conditions on the absorption spectra of BG1189 and BG1190.

For both quinazoline derivatives, normal room lighting conditions influence their spectral properties. BG1189 and BG1190 are unstable if they are not kept in dark, even in the first 24 h after preparation.

The only absorption spectrum similar to the one of the fresh prepared solution of BG1189 is the spectrum of the irradiated solution at 355 nm for 2 min, with an average energy per pulse E = 6 mJ. Consequently, the total energy absorbed by solution in 2 min was not enough to modify a significant fraction of the molecules. Even though the total energy emitted in 2 min was the same for both wavelengths (E_total_ = 7200 mJ, considering E = 6 mJ and pulse frequency 10 pps), the BG1189 solution was modified in the first 2 min when exposed to laser radiation, at λ = 266 nm. 

Two explanations can support the rapid modification at λ = 266 nm: first, the molar extinction coefficient (ε) for BG1189 calculated from the absorption spectra was higher at 266 nm (ε_266nm_ = 4866 L mol^−1^ cm^−1^) than at 355 nm (ε_355nm_ = 3060 L mol^−1^ cm^−1^), so that BG1189 solution absorbs more at 266 nm, and, secondly, the photon energy is larger at 266 nm (E_ph266nm_ = 449.7 kJ/mol) than at 355 nm (E_ph355nm_ = 336.9 kJ/mol) and it is more likely to break more chemical bonds at λ = 266 nm.

For BG1190 photostability study, spectra of solutions exposed for 2 min and for 5 min to laser radiation at λ = 355 nm remain in the error limits, indicating that the solutions remain stable in these conditions (Figure 5b) for the same reasons as in the case of BG1189; for BG1190 solution, ε_266nm_ is 3855 L mol^−1^ cm^−1^ and ε_355nm_ is 2800 L mol^−1^ cm^−1^. The lower value of ε_355nm_ explains why more than 5 min of irradiation are needed to modify the BG1190 solutions, whereas in the case of BG1189 less than 5 min of exposure to laser radiation at 355 nm are enough. 

For longer periods of irradiation, modifications of the absorption spectra are obvious. Due to higher absorbance at 266 nm, quinazoline derivatives start to modify rapidly (before 2 min) when exposed to laser radiation having this wavelength. 

**Figure 6:**
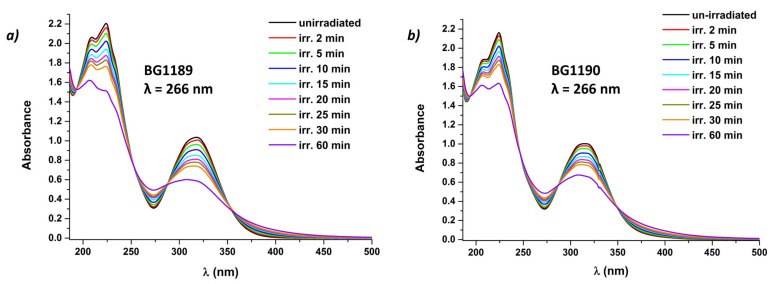
Laser induced modifications of the absorption spectra of: (**a**) BG1189 and (**b**) BG1190. Solutions prepared in ultrapure water at 10^−3^ M. Laser irradiation conditions: E = 6 mJ, λ = 266 nm.

Figure 6 presents the absorption spectra of BG1189 and BG1190 irradiated for different periods (up to 60 min). The hypochromic effect observed during irradiation indicates a modification of the compounds. Moreover, a hypsochromic shift was observed from 318 nm to 307 nm for BG1189 (Figure 6a) and from 314 nm to 308 nm in the case of BG1190 (Figure 6b).

Considering that the absorption band with the peak at 224 nm is due to π–π* transition, and the bands at 318/314 nm are attributed to *n*–π* transition (as resulted from the blue shift obtained when changed to a more polar solvent), these bands being characteristic for the quinazoline radical [38], the hypochromic effect and the hypsochromic shift indicate that the quinazoline radical of both derivatives was modified during irradiation.

In Figure 6a, three isosbestic points are detected at 251, 288 and 352 nm. In Figure 6b, there are also three isosbestic points, at 246, 288 and 348 nm. The existence of an isosbestic point reflects the presence of two chemical species in the solution [41]. Considering that it is less likely to have two chemical species with the same ε at three different wavelengths, Figure 6 shows that after 60 min of irradiation it is possible to generate at least six photoproducts for each tested quinazoline.

When BG1189 and BG1190 solutions are exposed to laser radiation at λ = 355 nm, E = 6 mJ, the curves are similar to the ones in Figure 6. It can be noted that hypochromic effect is somewhat smaller because the absorbance is smaller at 355 nm than at 266 nm and the modifications of molecules do not occur as rapidly. Another difference would be that the isosbestic points appear at 248, 290 and 351 nm for BG1189 and at 247, 289 and 347 nm for BG1190.

#### 2.3.2. FTIR Spectroscopy

The frequencies of bands obtained for the non-irradiated BG1189 solution from the recorded FTIR spectrum were compared with the theoretical frequencies calculated with Gaussian 09 and GaussView 5.0 (see Table S1 from Annex 1) [42]. Similar data are given for BG1190 in Table S2 from Annex 1.

Quinazolines have strong IR absorption bands between 1635–1610 cm^−1^, 1580–1565 cm^−1^ and 1520–1475 cm^−1^, and other bands with variable intensity in the range 1500–1300 cm^−1^, all originating from aromatic ring vibrations. They also display IR absorption bands between 1290 and 1010 cm^−1^, due to C–H in-plane deformation vibrations; between 1000 and 700 cm^−1^ they have bands originating from the C–H out-of-plane deformation vibrations [43].

**Figure 7:**
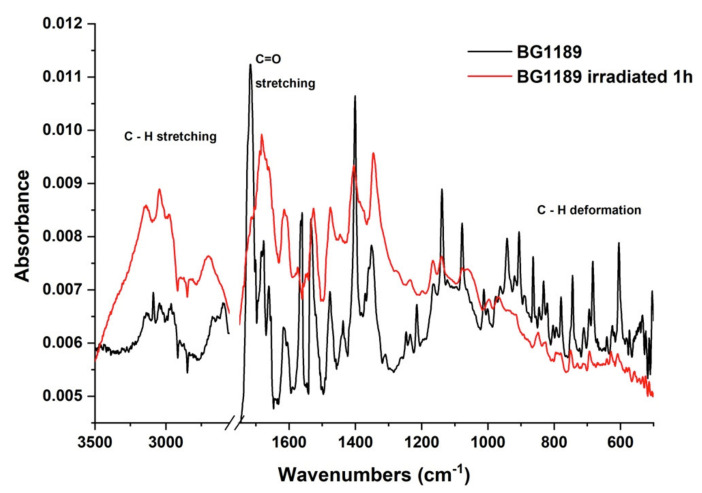
FTIR spectra of the unirradiated and 1 h irradiated BG1189 10^−3^ M solutions in water.

The FTIR spectra recorded for the BG1189 solutions in water before and after exposure to laser radiation are given in Figure 7.

The FTIR spectrum of the BG1189 exposed one hour to laser radiation (λ = 266 nm; E = 6 mJ) presents modifications compared to the unirradiated sample. Absorption bands with increased intensity may be observed with the maxima at 3140, 3044, 2979 and 2707 cm^−1^. This modification suggests an increase of CH_2_ asymmetrical and symmetrical stretching vibrations. The disappearance of the 1716 cm^−1^ peak and the formation of a new wide absorption band with maximum at 1683 cm^−1^ indicates the disruption of the majority of C=O bonds and an increase of C–C and C–N stretching vibrations from quinazoline radical, or just a superposition of the remaining C=O stretching vibrations with the remaining C–C and C–N stretching vibrations leading to appearance of new weaker and wider absorption ban. The absence of the 1561 cm^−1^ peak in the spectrum of the irradiated BG1189 shows a decrease of C–C ring stretching, N–O stretching, O–H deformation and C–H deformation vibrations suggesting disruption of some of these bonds. A decrease in intensity of the 1401 cm^-1^ band indicates a diminution of C–H deformation and C–N stretching vibrations. The disappearance of the absorption band with maximum at 1214 cm^−1^ and the reduction of the ones at 1138 and 1078 cm^−1^ point to a reduction of C–C and C–N ring stretching vibrations, C–H and O–H deformation vibrations and CH_2_ wagging vibrations from CH_3_ groups. After exposure to laser radiation for one hour, the IR absorption bands observed for BG1189 sample in the range 1100–450 cm^−1^, either disappear, or their intensity is extremely reduced, indicating a decrease of C–C and C–N stretching vibrations from the quinazoline ring, N–O stretching or deformation vibrations, O–H and C=O deformation vibrations and all types of C–H deformation vibrations.

All these modifications observed in IR spectrum of the irradiated BG1189 in comparison with the spectrum of the unirradiated sample suggest that laser radiation breaks part of the chemical bonds, affecting not only the substituents, but also the quinazoline radical of BG1189 molecules.

**Figure 8:**
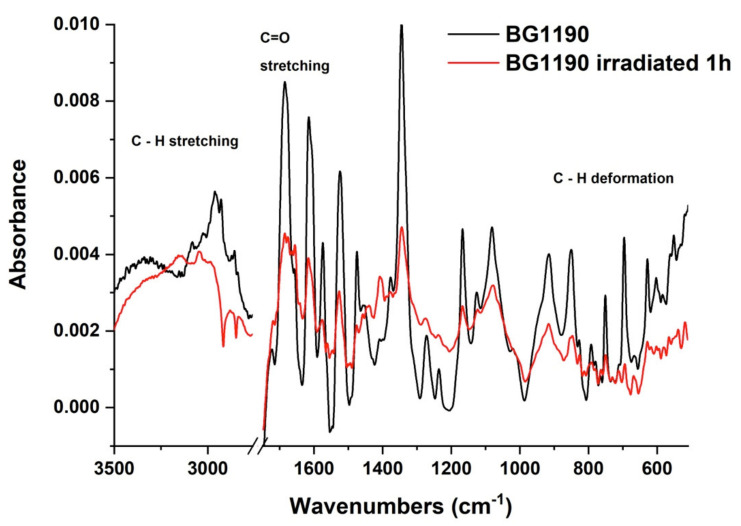
FTIR spectra of the unirradiated and 1 h irradiated BG1190 10^−3^ M solutions in water.

The FTIR spectrum of the irradiated solutions of the BG1190 at 10^−3^ M in water, shown in Figure 8, displays modifications in comparison to the spectrum of the unirradiated BG1190. After exposure to laser radiation (λ = 266 nm; E = 6 mJ; t = 1 h), the IR absorption bands in the range 3100–2800 cm^−1^ decreased in intensity, indicating the breaking of the majority of CH bonds, which were performing CH_2_ symmetrical and asymmetrical stretching vibrations. The band with maximum at 1685 cm^−1^ diminishes to approximately half of its intensity after irradiation, suggesting that the number of C=O stretching vibrations, hence the number of carbonyl groups, is reduced by half. Larger decreases in intensity are observed for the bands with maxima at 1615, 1574, 1524 and 1474 cm^−1^, indicating a decrease of C–C and C–N stretching vibrations from the quinazoline radical, N–O stretching vibrations, C–N deformation vibrations, O–H deformation vibrations, C–H deformation vibrations from quinazoline ring and CH_2_ scissoring. Reduction in intensity of the peak at 1345 cm^−1^ suggests a diminished number of N–O stretching, O–H deformation and CH_2_ twisting vibrations. The intensity decrease of the absorption bands with maxima at 1167, 1126 and 1081 cm^−1^ hints about the breaking part of the C–C, C–N and C–H bonds from the quinazoline ring and N–O, C–C and C–N bonds from the substituents. The range 1000–400 cm^−1^ displays bands that also have reduced intensity compared to the FTIR spectrum of the unirradiated sample. This suggests a diminution of the following vibrations: C–C, C–N and C–H deformation and stretching from quinazoline radical, N–O and NO_2_ deformation, along with CH_2_ rocking and wagging vibrations.

Similar to BG1189 samples, FTIR spectra recorded before and after exposure to laser radiation show that BG1190 molecules are modified during irradiation, part of the chemical bonds being braked in the substituents and in the aromatic rings. 

## 3. Materials and Methods

### 3.1. Materials

Due to the fact that quinazoline derivatives have a wide range of biomedical activities and applications, including anti-bacterial, anti-cancer, anti-inflammatory, analgesic, anti-virus, anti-tuberculosis, anti-malarial effect, some of them were chosen as potential chemosensitizers [44].

The quinazoline derivatives, labelled BG1189 and BG1190, studied in this paper were designed as EPIs at UMR-MD1, Facultés de Médecine et de Pharmacie, Université de la Méditerranée, Marseille, France. The chemical synthesis of these derivatives was reported in [45]. 

**Figure 9:**
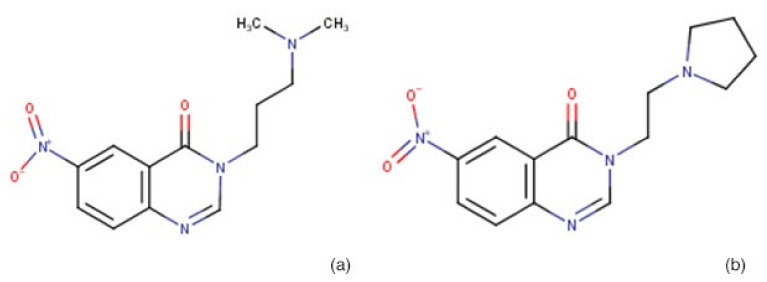
Chemical structures of quinazoline derivatives: (**a**) BG1189 and (**b**) BG1190.

The molecular formulas of BG1189 and BG1190 are C_13_H_16_N_4_O_3_ and C_14_H_16_N_4_O_3_, respectively. Their chemical structures are shown in Figure 9.

### 3.2. Methods

For molecular docking assay we have used Autodock 4.2.6 [46]. Using Open Babel we have converted BG1189 and BG1190 molecules optimized initially with Gaussian 09 in pdbqt format [47]. The 3D structure of the membrane protein was imported from RCSB Protein Data Bank [48] in PDB format. We kept only subunit AcrB chain B for AcrB (PDB code: 5NC5) for molecular docking protocol [23]. We have used our molecular docking protocol to simulate the interaction between quinazoline derivatives in target protein [29].

We set the specific grid points (x, y, z) at 50, the grid point spacing at 0.05 nm and the coordinates of the central grid point of maps (x, y, z) 30.547, −51.785, −51.989.

The solutions of BG1189 and BG1190 were prepared in ultrapure deionized water, prepared with a filtering system described in [49]. The concentration of both quinazoline solutions was 10^−3^ M and it was chosen according to the biological activity assessments. For each compound, six samples were prepared in total, out of which three were kept in dark at 4, 22 and 37 °C, respectively, one was kept at 22 °C and at normal illumination of the room, and two were exposed to laser radiation at two different wavelengths immediately after the solution was prepared.

The stability studies were performed comparing the UV-Vis absorption spectra obtained at different times after preparation for samples kept at usual storage and body temperatures (4, 22 and 37 °C) and in different illumination conditions: dark, normal room lighting, exposed to laser radiation. UV-Vis absorption spectra were measured using a Perkin Elmer Lambda 950 UV/Vis/NIR spectrophotometer, with an error of ± 0.004%. The spectra were recorded between 200 and 800 nm, using as reference an optical cell containing the solvent, either ultrapure deionized water or DMSO. The thickness of the optical cells used for absorption spectra measurements was 1 mm, which may cause measuring errors due to the positioning of the cell in the sample holder of the spectrophotometer. For this, 20 repetitive measurements were performed, repositioning each time the optical cell. The error is given as a percent of the absorbance values and was calculated as the difference between the highest and lowest intensities of the absorption peak obtained in the 20 repetitive measurements. The spectra were recorded every hour in the first day after solutions’ preparation and then every 24 h for the next month (624 h). The method of measuring and calculating the experimental errors was fully described in [37]. The graphs were made by superimposing the recorded spectra in Origin 2019 (9.6) software, without any preprocessing.

Two types of photostability studies were performed: first, to assess for how long the solutions of quinazoline derivatives can be kept in dark at normal room illumination without changing their properties; secondly, to determine the radiation dose needed to obtain photoproducts with enhanced antimicrobial activity in comparison with the initial/parent molecules.

For the photostability study, three samples of each quinazoline derivative solutions in ultrapure water, with a concentration of 10^−3^ M, were prepared. One was stored at normal room lighting for 624 h and two were exposed to laser radiation, with average pulse energy E = 6 mJ, at wavelengths 266 and 355 nm, respectively.

The laser radiation used to irradiate the solutions was provided by the third harmonic generation (THG) at 355 nm and fourth harmonic generation (FHG) at 266 nm of a Nd:YAG (Continuum, Excel Technology) pulsed laser, model Surelite II with 10 Hz pulse repetition rate and 6 ns pulse duration. The average beam energy was 6 mJ, at both wavelengths.

In addition to UV-Vis absorption spectroscopy, FTIR spectroscopy was employed to evaluate the photostability of the quinazoline derivatives solutions.

FTIR spectra of solutions before and after exposure to laser radiation were recorded with a FTIR spectrometer Nicolet iS50 (Thermo Scientific, Waltham, MA, USA). To acquire the spectra, a 40 µL sample was placed on a KRS-5 (Thallium Bromo-Iodide) optical crystal and dried before recording their absorbance to minimize the influence of water. The recorded FTIR spectra were smoothed with Origin 2019 (9.6) software by Savitzky–Golay 25 points method.

The recorded FTIR spectra of the quinazoline derivatives were compared to the ones calculated by Gaussian 09 and GaussView 5.0., using the method described in [50]. Density functional theory (DFT) with the B3LYP (Becke, 3-parameter, Lee-Yang-Parr) method and 6-311G(d,p) basis set were employed for calculating the vibrational frequency of the BG1189 and BG1190 molecules. The calculations reported in this paper were performed for the quinazoline derivatives solvated in water.

## 4. Conclusions

This paper presents the pharmacokinetic and pharmacodynamic profiles, along with a spectral characterization of two quinazoline derivatives, labelled BG1189 and BG1190, designed as potential EPIs.

Both derivatives are good candidates as drugs since they follow all the applied drug-like rules (Table 1). ADMET predictions illustrate that these derivatives had a high absorption (if administrated orally), are well distributed in tissues, and have relatively low toxicity, compared to clinically used drugs (Table 2). 

Molecular docking simulations showed that both compounds present a good inhibitory activity on subunit AcrB chain B. Additionally, the low LogP values recommend these derivatives as promising antibacterial drugs (Table 1 and Table 3). Previous studies demonstrated the affinity of the tested compounds. Firstly, minimum inhibitory concentrations (MIC) of the compounds have been assayed on a strain that overexpressed AcrAB pump (CM64, an ATCC 13048 derivative), the parental wild type strain, and several clinical isolates that overexpressed AcrAB pump. Results indicated that the quinazoline derivatives had no activity on AcrAB-deleted strain. Secondly, the compounds significantly increased the accumulation of radiolabeled chloramphenicol, a well-known substrate of AcrAB pump, in strains overexpressing AcrAB. Moreover, the same study showed a variation of ligands location inside the AcrB cavity [16]. We obtained similar results in our theoretical model as we presented in Figure 1.

Given the above data and previous tests on bacteria, these quinazoline derivatives represent suitable candidates in future experiments as antimicrobial agents, and further affinity tests on AcrAB-TolC are recommended [16].

Spectral characterization provided an insight into the stability and photostability of the studied compounds.

UV-Vis absorption spectroscopy allowed to determine the stability of quinazoline solutions in water or in DMSO, in constant or in variable environmental conditions. The photostability of the samples was assessed through UV-Vis absorption spectroscopy and FTIR spectroscopy.

The UV-Vis absorption spectra of BG1189 and BG1190 in water, at 10^−3^ M concentration, kept in dark, at 22 °C (room temperature), remained in the limits of error, showing that the samples were stable for 624 h. The solutions prepared in DMSO, kept in similar conditions, remained stable only 552 h after preparation in the case of BG1189 and 216 h in the case of BG1190. 

A comparison between UV-Vis absorption spectra of samples from both quinazoline derivatives, kept at 4 °C (refrigerator temperature), 22 °C (room temperature), and 37 °C (body temperature), showed that the most stable samples were the ones kept at room temperature, indicating 22 °C as best fitted storage temperature.

The photostability study identified first the illuminations conditions needed to keep the quinazoline derivatives solutions unaltered. Afterwards, illumination conditions were varied (from ambient light to laser radiation exposure) so that we could determine the modifications of the quinazoline derivatives and the generation of photoproducts.

FTIR spectra of BG1189 and BG1190 recorded before and after irradiation, demonstrated that both derivatives were modified during laser exposure, suffering alterations of the substituents and of the aromatic rings.

Further studies need to be conducted for a better understanding of the modifications, but current data allowed us to have an insight on the stability of these compounds proposed as EPIs.

## Figures and Tables

**Table 1:** Drug-like rules Lipinski, Ghose and Veber validation for molecules BG1189 and BG1190.

Molecule	BG1190			BG1189		
	Validate	Molecular Descriptors	Value	Validate	Molecular Descriptors	Value
Lipinski	Yes	MW ≤ 500	289.31	Yes	MW ≤ 500	277.30
		MlogP*_o/w_* ≤ 4.15	1.04		MlogP*_o_*_/*w*_ ≤ 4.15	0.37
		Acc ≤ 10	5		Acc ≤ 10	5
		Don ≤ 5	1		Don ≤ 5	1
Ghose	Yes	160 ≤ MW ≤480	289.31	Yes	160 ≤ MW ≤ 480	277.30
		−0.4 ≤ WlogP*_o/w_* ≤ 5,6	0.91		-0.4 ≤ WlogP*_o/w_*≤ 5,6	1.15
		40 ≤ MR ≤ 130	81.07		40 ≤ MR ≤ 130	74.46
		20 ≤ atoms ≤ 70	38		20 ≤ atoms ≤ 70	37
Veber	Yes	Rotatable bonds ≤ 10	4	Yes	Rotatable bonds ≤ 10	5
		TPSA ≤ 140 Å^2^	78.44 Å^2^		TPSA ≤ 140 Å^2^	78.44 Å^2^

**Table 2:** Predicted ADMET results for BG1189 and BG1190 quinazoline derivatives compared with predicted ADMET values of norfloxacin.

Model Name	Predicted Value BG1190	Predicted Value BG1189	Predicted Value Norfloxacin	Unit
Water Solubility	−1.992	−1.549	−3.176	log mol/L
Caco2 Permeability	0.76	0.597	1.242	log Papp in 10^−6^ cm/s
Intestinal Absorption (Human)	87.391	78.106	86.904	% Absorbed
VDss (Human)	0.909	0.84	0.064	log L/kg
Fraction Unbound (Human)	0.713	0.612	0.462	Fu
Total Clearance	0.811	0.594	0.366	log ml/min/kg
Renal OCT2 Substrate	Yes	No	No	Yes/No
hERG I Inhibitor	No	No	No	Yes/No
hERG II Inhibitor	Yes	Yes	No	Yes/No
Oral Rat Acute Toxicity (LD50)	2.55	2.541	2.217	mol/kg
Hepatotoxicity	Yes	Yes	Yes	Yes/No

**Table 3:** Molecular docking results for quinazoline derivatives BG1189 and BG1190 on multidrug efflux pump subunit AcrB (PDB code: 5NC5). AA residues in h-bound with quinazoline derivatives are underlined.

Multidrug Efflux Pump Subunit AcrB (PDB Code: 5NC5)	
AA Residues from Allosteric Situs of AcrB Subunit.	Met573; Phe617; Phe628; Phe666; Leu668.
Lowest EFEB BG1190	−8.21 kcal/mol
AA from the Run with Lowest EFEB of BG1990	Gln34; Pro36; Thr37; Ala39; Ser134; Ser135; Phe136; Tyr327; Thr329; Leu668; Ile671; Val672; Glu673.
Lowest EFEB BG1189	−7.40 kcal/mol
AA from the Run with Lowest EFEB of BG1989	Gln34; Tyr325; Pro326; Tyr327; Asp328; Thr329; Pro331; Phe332; Gly570; Val571; Gln569; Glu607; Ser608; Ser630; Leu631; Trp634; Leu668.

## Data Availability

Data available on request.

## Supplementary Materials

The following are available online, Annex 1—FTIR spectroscopy.
