# Supplementary material for: Quinazoline Derivatives Designed as Efflux Pump Inhibitors: Molecular Modeling and Spectroscopic Studies
Source: Molecules. 2021 Apr 19;26(8):2374. doi: 10.3390/molecules26082374 (PMC8073189; doi:10.3390/molecules26082374)
Supplement: Supplementary file 1 [file molecules-26-02374-s001.zip › molecules-1182818-supplementary.pdf]

Annex 1 – FTIR spectroscopy

The frequencies of bands obtained for the non-irradiated BG1189 solution in the recorded FTIR spectrum were compared with the theoretical frequencies calculated with Gaussian 09 and GaussView 5.0.

Table S1. Experimental and calculated vibration frequencies for BG1189.

Observed frequencies (cm^{-1})	Calculated frequencies (cm^{-1})	Assigned vibrations
-	3729	O–H stretching
3152, 3138, 3129, 3122	3146, 3129, 3116, 3111	CH_2 asymmetrical stretching
3088, 3045, 3033, 3017	3086, 3082, 3070, 3058	CH_2 symmetrical stretching
2977, 2962	2939, 2931	C–H stretching from CH_3 groups
1716	1731	C=O stretching
1677, 1660	1652, 1629	C–C stretching and C–N stretching from quinazoline radical
1616	1617	C–C ring stretching; N–O stretching; O–H deformation
1561	1574	C–C ring stretching; N–O stretching; O–H deformation; C–H deformation
1533	1522	C–H deformation
1477	1504	C–C ring stretching; N–O stretching; O–H deformation; C–H deformation
1437	1428	C–H deformation; CH_2 wagging
1401, 1382	1410, 1407	C–H deformation, C–N stretching
1370	1381	C–C ring stretching; C–H deformation; O–H deformation
1351	1331	N–O stretching; O–H deformation
1310	1324	C–H deformation
1246	1300	C–N ring stretching; C–H deformation from CH_3 groups; C–H deformation; O–H deformation
1214	1262	C–C ring stretching; C–N ring stretching; C–H deformation; O–H deformation
1163	1217	C–C ring stretching; C–N stretching; O–H deformation; C–H deformation
1138	1199	CH_2 wagging from CH_3 groups; C–H deformation
1078	1161	C–N ring stretching; C–H deformation; CH_2 wagging from CH_3 groups
1011	1118	CH_2 wagging from CH_3 groups; C–H deformation; C–C ring stretching; C–N stretching; N–O stretching; O–H deformation
962, 942	1019, 999	C–C ring stretching; C–N ring stretching; C–H deformation; O–H deformation; N–O stretching; CH_2 wagging from CH_3 groups
919	910	C–C ring stretching; C–N ring stretching; C–H deformation; N–O stretching; CH_2 rocking
906	901	CH_2 rocking
890	872	CH_2 twisting; CH_2 rocking
863	852	C–N stretching; CH_2 rocking
832	803	C–C ring stretching; C–N ring stretching; C–H deformation; O–H deformation; C=O deformation
779	792	C–C ring deformation; C–N ring deformation; C–H deformation; C=O deformation; CH_2 rocking
744	744	C–C ring stretching; C–N ring stretching; C–H deformation; O–H deformation; CH_2 rocking
683, 605	688, 572	C–C ring deformation; C–N ring deformation; N–O deformation; O–H deformation; CH_2 rocking
533, 504	553, 511	C–C ring deformation; C–N ring deformation; N–O deformation; O–H deformation; C–H deformation; CH_2 wagging

477	483	C–C ring deformation; C–N ring deformation; N–O deformation; O–H deformation; CH_2 wagging

The FTIR spectrum of the unirradiated BG1189 evidenced bands between 3200 cm^{-1} and 2950 cm^{-1} that can be assig symmetrical and asymmetrical stretching vibrations of C–H bonds. The strongest vibration is the one of the C=O bond and i sponsible for the band with the maximum at 1716 cm^{-1}. C–C and C–N stretching vibrations from quinazoline radical are resp for the absorption bands with maxima at 1677 cm^{-1} and 1661 cm^{-1}. C–C stretching from the aromatic rings, N–O stretching an deformation vibrations contribute to the appearance of the peaks at 1616 cm^{-1}, 1561 cm^{-1} and 1477 cm^{-1}, respectively. C–H deform also contribute to the last two peaks. The peak observed at 1533 cm^{-1} is due to C–H bending vibrations and the one at 1437 cm^{-1} to C–H bending and CH_2 wagging vibrations. Strong C–N stretching vibrations along with C–H deformation vibrations give r high intensity absorbance band with maximum at 1401 cm^{-1} and a weaker one with maximum at 1382 cm^{-1}. N–O stretching an deformation vibrations are responsible for the peak at 1351 cm^{-1}. A weak absorption band due to C–H deformation vibrat observed in the IR spectrum with the peak at 1310 cm^{-1}. C–N ring stretching vibrations, C–H deformation vibrations from CH_3 g C–H deformation and O–H deformation vibrations give rise to the peak at 1246 cm^{-1}. C–C and C–N ring stretching vibration and O–H deformation vibrations are responsible for the appearance of the IR band with maximum at 1214 cm^{-1}. The peak at 11 ^{1} is due to similar vibrations as the previous one, except C–N stretching vibrations that are not originating from the aromati but from the substituents bonded to the quinazoline radical. CH_2 wagging vibrations from CH_3 groups and C–H deformation tions give rise to the absorption band with maximum at 1138 cm^{-1}. Along with the vibrations responsible for the previous pea ring stretching vibrations also contribute for the appearance of the 1078 cm^{-1} peak. The peak at 1011 cm^{-1} is due to CH_2 waggin CH_3 groups, C–H deformation, C–C ring stretching, C–N stretching, N–O stretching, O–H deformation vibrations. The same tions are responsible for the 962 cm^{-1} and 942 cm^{-1} peaks, except that the C–N stretching vibrations originate in the quinazoline r The absorption band with maximum at 919 cm^{-1} is due to C–C and C–N ring stretching, C–H deformation, N–O stretching ar rocking vibrations. The peak at 906 cm^{-1} appears due to CH_2 rocking vibrations. These vibrations together with the CH_2 tv vibrations contribute to the appearance of the absorption band with the peak at 890 cm^{-1}. The peak at 863 cm^{-1} appears due t stretching and CH_2 rocking vibrations and the one at 832 cm^{-1} is due to C–C ring and C–N ring stretching vibrations and C–H and C=O deformation vibrations. C–C and C–N ring deformation, C–H and C=O deformation and CH_2 rocking vibrations give the peak at 779 cm^{-1}. The peak at 744 cm^{-1} appears due to C–C and C–N ring stretching vibrations, C–H and O–H deformati CH_2 rocking vibrations. The absorption bands with maxima at 683 cm^{-1} and 604 cm^{-1} originate from the C–C and C–N ring deforr N–O and O–H deformation and CH_2 rocking vibrations. C–C and C–N ring deformation, N–O, O–H and C–H deformation ar wagging vibrations contribute to the formation of the peaks at 533 cm^{-1} and 504 cm^{-1}; the same vibrations, except C–H deforr give rise to the peak 477 cm^{-1}.

A comparison between the experimental IR frequencies and the calculated ones, along with an assignation of the vib responsible for each absorption band in the FTIR spectrum of the BG1190 solution, is given in Table S2.

Table S2. Experimental and calculated frequencies for BG1190.

**Observed frequencies (cm^{-1})**	**Calculated frequencies (cm^{-1})**	**Assigned vibrations**
-	3729	O–H stretching
3084, 3024, 2964, 2928	3160, 3122, 3104, 3098	CH_2 asymmetrical stretching
2872, 2857, 2840	3060, 3046, 3033	CH_2 symmetrical stretching
1685	1731	C=O stretching
1657	1652	C–C and C–N stretching and C-H deformation from quinazoline ring
1615	1616	C–C ring stretching; C–N deformation; N–O stretching and O–H deformation
1574	1574	C–C ring stretching; C–N deformation; N–O stretching; O–H deformation; C–H deformation from quinazoline ring
1524	1474	CH_2 scissoring; C–N and C–C ring stretching; C–H deformation from quinazoline ring; N–O stretching and O–H deformation
1474, 1457	1468, 1437	C–C stretching and C–H deformation from quinazoline ring; CH_2 scissoring

1428	1424	C–H deformation from quinazoline ring; CH_2 wagging
1409	1401	C–N stretching and C–H deformation from quinazoline ring; CH_2 twisting
1399	1381	C–C stretching and C–H deformation from quinazoline ring; CH_2 wagging;
1376	1377, 1362	CH_2 wagging
1345	1334, 1329	N–O stretching and O–H deformation; CH_2 twisting
1311	1300	C–C, C–N and C-H deformation from quinazoline ring; C–C and C–N stretching from quinazoline ring; N–O and O–H deformation
1271	1277, 1270	C–C and C–N stretching from quinazoline ring; N–O stretching; C–H deformation from quinazoline ring; CH_2 twisting
-	1247	C–C, C–N and C-H deformation from quinazoline ring; C–N stretching; CH_2 twisting
1235	1222	C–C, C–N and C-H deformation from quinazoline ring; C–N stretching; CH_2 twisting; N–O stretching
-	1213	C–C, C–N and C-H deformation from quinazoline ring; C–N stretching; CH_2 rocking; N–O stretching
1167	1142	C–C, C–N and C–H deformation from quinazoline ring; C–N stretching from quinazoline ring; CH_2 twisting
1126	1113	C–C, C–N and C–H deformation from quinazoline ring; N–O deformation; CH_2 rocking
1081	1082	C–C and C–N deformation; CH_2 twisting from radical ring
1020	1023	C-H deformation from quinazoline ring; C–C and C–N deformation and CH_2 rocking from radical ring
1009	1000	NO_2 stretching; C–C, C–N and C-H stretching from quinazoline ring
941	947	C-H deformation from quinazoline ring; CH_2 rocking
915	906	C–C and C–N stretching from quinazoline ring; NO_2 stretching; CH_2 rocking
899	902	C–H deformation from quinazoline ring
-	877	C–C and C–N deformation from radical ring; CH_2 deformation
851	851	C–C, C–N and C-H ring deformation
827, 792, 779, 750	816, 802, 792, 767	C–C and C–N ring deformation; CH_2 rocking
729	745	C–C and C–H ring stretching; NO_2 deformation; CH_2 rocking
695	689	C–C and C–N ring deformation; NO_2 deformation; CH_2 rocking
627	571	NO_2 scissoring; C–C and C–N ring deformation; CH_2 wagging
551	555, 542	C–C and C–N ring deformation; CH_2 wagging and rocking
503	506	N–O deformation
480	484	C–C ring deformation; N–O deformation; CH_2 wagging
465, 453, 418	414, 412	C–N ring deformation; N–O deformation; CH_2 wagging

The FTIR spectrum of the BG1190 solution, showed in Figure 9, presents absorption bands with peaks at 3084 cm^{-1}, 302 2964 cm^{-1} and 2928 cm^{-1}, which can be assigned to CH_2 asymmetrical stretching vibrations. CH_2 symmetrical stretching vibrati responsible for the appearance of the IR absorption bands with maxima at 2872 cm^{-1}, 2857 cm^{-1} and 2840 cm^{-1}. A strong absorptio is observed at 1685 cm^{-1} originating from C=O stretching vibrations. C–C and C–N stretching vibrations and C-H deformation vib from quinazoline ring give rise to the peak at 1657 cm^{-1}. The peak at 1615 cm^{-1} is due to C–C ring stretching, C–N deformatio stretching and O–H deformation vibrations. These four types of vibrations along with C–H deformation vibrations from the qu line ring are responsible for the peak at 1574 cm^{-1}. The peak at 1524 cm^{-1} appears due to CH_2 scissoring vibrations, C–N and C- stretching vibrations, C–H deformation vibrations from quinazoline ring, N–O stretching and O–H deformation vibration stretching vibrations and C–H deformation vibrations from quinazoline ring, along with CH_2 scissoring vibrations lead to for of the absorption bands with maxima at 1474 cm^{-1} and 1457 cm^{-1}. C–H deformation vibrations from quinazoline ring superin with CH_2 wagging vibrations are responsible for the appearance of the peak at 1428 cm^{-1}. The peak at 1409 cm^{-1} originates from N stretching, and C–H deformation vibrations from quinazoline ring and CH_2 twisting vibrations. The absorption band with th imum at 1399 cm^{-1} appears due to C–C stretching and C–H deformation vibrations from quinazoline ring and CH_2 wagging vibr

Last type of vibrations is also responsible for the peak at 1376 cm^{-1}. A strong absorption band with the maximum at 1345 cm^{-1} [illegible] assigned to N–O stretching, O–H deformation and CH_2 twisting vibrations. C–C, C–N and C-H deformation vibrations from qu[illegible] line ring, C–C and C–N stretching vibrations from quinazoline ring, along with N–O and O–H deformation vibrations are resp[illegible] for the appearance of the shoulder at 1311 cm^{-1}. The peak observed at 1235 cm^{-1} appears due to C–C, C–N and C-H deformation [illegible] tions from quinazoline ring, C–N and N–O stretching vibrations, together with CH_2 twisting vibrations. C–C, C–N and C-H [illegible] mation vibrations and C–N stretching vibrations from quinazoline ring along with CH_2 twisting give rise to the peak at 1167 cm[illegible] IR band with maximum at 1126 cm^{-1} appears due to C–C, C–N and C-H deformation vibrations from quinazoline ring, N–O [illegible] mation and CH_2 rocking vibrations. C–C and C–N deformation vibrations together with CH_2 twisting vibrations from the ar[illegible] rings are responsible for the peak at 1081 cm^{-1}. The peak at 1020 cm^{-1} originates from the superposition of the following vibrati[illegible] H deformation and CH_2 rocking vibrations from quinazoline ring along with C–C and C–N deformation vibrations. The abso[illegible] band with the maximum at 1009 cm^{-1} is due to NO_2 stretching vibrations in combination with C–C, C–N and C-H stretching vib[illegible] from quinazoline ring. C–H deformation vibrations from the aromatic rings and CH_2 rocking vibrations lead to formation of th[illegible] with maximum at 941 cm^{-1}. The peak at 915 cm^{-1} appears due to C–C and C–N stretching vibrations from quinazoline ring, NO_2 s[illegible] ing vibrations and CH_2 rocking vibrations. C–H deformation vibrations from the quinazoline radical are responsible for the b[illegible] 899 cm^{-1} and C–C, C–N and C–H deformation vibrations, from the quinazoline ring as well, give rise to the peak at 851 cm^{-1}. C[illegible] C–N ring deformation vibrations along with CH_2 rocking vibrations lead to the formation of consecutive absorption bands with m[illegible] at 827 cm^{-1}, 792 cm^{-1}, 779 cm^{-1} and 750 cm^{-1}. The peak at 729 cm^{-1} originates from C–C and C–H quinazoline ring stretching vibr[illegible] NO_2 deformation vibrations along with CH_2 rocking vibrations. The peak at 695 cm^{-1} appears due to C–C and C–N deformation [illegible] tions from the aromatic radical, NO_2 deformation and CH_2 rocking vibrations. NO_2 scissoring vibrations, C–C and C–N defor[illegible] vibrations from the quinazoline ring along with CH_2 wagging vibrations give rise to an absorption band with maximum at 62[illegible] The peak at 551 cm^{-1} is due to C–C and C–N deformation vibrations from the quinazoline radical together with CH_2 waggi[illegible] rocking vibrations. N–O deformation vibrations are responsible for the appearance of the absorption band with maximum at 5[illegible] and C–C ring deformation vibrations, N–O deformation vibrations along with CH_2 wagging vibrations. C–N deformation vib[illegible] from the quinazoline ring, N–O deformation vibrations and CH_2 wagging vibrations give rise to absorption bands with maxima [illegible] cm^{-1}, 453 cm^{-1} and 418 cm^{-1}.
